# Knowledge-Based Recurrent Neural Network for TCM Cerebral Palsy Diagnosis

**DOI:** 10.1155/2022/7708376

**Published:** 2022-10-12

**Authors:** Dongmei Li, Jintao Qu, Ziwei Tian, Zijun Mou, Lei Zhang, Xiaoping Zhang

**Affiliations:** ^1^School of Information Science and Technology, Beijing Forestry University, Beijing 100083, China; ^2^Engineering Research Center for Forestry-oriented Intelligent Information Processing, National Forestry and Grassland Administration, Beijing 100083, China; ^3^Shandong University of Traditional Chinese Medicine, Jinan 250355, Shandong, China; ^4^National Data Center of Traditional Chinese Medicine, China Academy of Chinese Medical Sciences, Beijing 100700, China

## Abstract

Cerebral palsy is one of the most prevalent neurological disorders and the most frequent cause of disability. Identifying the syndrome by patients' symptoms is the key to traditional Chinese medicine (TCM) cerebral palsy treatment. Artificial intelligence (AI) is advancing quickly in several sectors, including TCM. AI will considerably enhance the dependability and precision of diagnoses, expanding effective treatment methods' usage. Thus, for cerebral palsy, it is necessary to build a decision-making model to aid in the syndrome diagnosis process. While the recurrent neural network (RNN) model has the potential to capture the correlation between symptoms and syndromes from electronic medical records (EMRs), it lacks TCM knowledge. To make the model benefit from both TCM knowledge and EMRs, unlike the ordinary training routine, we begin by constructing a knowledge-based RNN (KBRNN) based on the cerebral palsy knowledge graph for domain knowledge. More specifically, we design an evolution algorithm for extracting knowledge in the cerebral palsy knowledge graph. Then, we embed the knowledge into tensors and inject them into the RNN. In addition, the KBRNN can benefit from the labeled EMRs. We use EMRs to fine-tune the KBRNN, which improves prediction accuracy. Our study shows that knowledge injection can effectively improve the model effect. The KBRNN can achieve 79.31% diagnostic accuracy with only knowledge injection. Moreover, the KBRNN can be further trained by the EMRs. The results show that the accuracy of fully trained KBRNN is 83.12%.

## 1. Introduction

Cerebral palsy is a leading cause of disability and could be challenging to cure throughout life [1]. The TCM theory plays an active role in the treatment of cerebral palsy. Symptoms are crucial in clinical diagnosis and treatment [2]. During clinical diagnosis, doctors integrate TCM theories to identify the syndrome based on patients' symptoms, which are heavily influenced by the doctor's previous experience. AI-assisted TCM diagnosis relies primarily on digital data obtained by modern electronic instruments, making TCM diagnosis more quantitative, objective, and standardized [3]. Thus, it is necessary to have a computer-aided decision-making model for the diagnosis to balance the uncertainty of human factors.

For the past two decades, owing to advancements in sensor, detector, and transducer technologies, it makes possible for AI to learn from digital information. Thus, AI-assisted TCM diagnosis has become a burgeoning field of research [4]. In earlier research, most AI approaches employed in TCM diagnosis are mostly limited to traditional machine-learning algorithms and their modified forms, such as support vector machine (SVM), random forest (RF), AdaBoost, and decision tree (DT). Wang [5] used a Bayesian classifier to generate the relationships between the human pulse and diagnostic. Zhang et al. [6] studied quantitative correlations between diseases and the physical appearance of the human tongue. In these conventional machine learning methods, the characteristics are extracted by specialists with extensive TCM clinical expertise. Deep learning technology has grown rapidly in recent years. Unlike the traditional machine learning methods, neurons in deep learning models can acquire diagnostic properties from the initial data set. The deep learning model comprises more complex hierarchical multilayer networks of artificial neurons that can automatically discover valuable features from the original data. Hu et al. [7] proposed a classifier by using the Shannon energy envelope, Hilbert transform, and deep convolutional neural networks (DCNN) for the analysis of the human pulse. Combing the characteristics of basic image processing and deep learning, Fu et al. [8] presented a computerized tongue coating nature diagnosis method using deep neural networks. Hou et al. [9] proposed a neural network for tongue color classification, which is more practical and accurate than the traditional one. Although the previous studies have attained a high level of accuracy, they only considered single-modal data and only a portion of patients' information. Therefore, recent studies are expected to introduce more comprehensive data. Yang et al. [10] developed a novel deep neural network that uses multiview features of the gene data to identify the disease genes. Dai et al. [11] proposed a multimodal deep learning framework based on the four-diagnosis of TCM. These approaches effectively compensate for the information in a single-modal and improve the accuracy of the model.

With the rise of medical digitalization, the hospital information system deposited a considerable volume of EMR data, which completely documents the patients' situation in text form. There is increasing interest in applying machine learning techniques to decision-making models for medical diagnosis and treatment. Liang et al. [12] adopted the deep belief network (DBN) to acquire feature representation from EMR and then combined the SVM for supervised learning on the labeled data. Similarly, various supervised machine learning algorithms such as random forest and logistic regression were used in [13] to build ischemic stroke classifiers. Although these ML-based methods outperform conventional techniques such as rule-based algorithms by using massive datasets, they ignore domain-specific knowledge.

The knowledge graph (KG), once known as ontology in early research, serves as an excellent solution to inject domain-specific knowledge into the ML models. The KG is a multirelational graph composed of entities and relationships containing a large amount of prior knowledge [14, 15]. Gone et al. [16] stood on advances in graph embedding learning techniques, decomposing the medicine recommendation task into a link prediction process, and proposed the safe medicine recommendation framework. Abdelaziz et al. [17] developed a large-scalesimilarly-based framework that predicts drug-drug interactions through text and graph embedding algorithms. These studies fully exploit the domain knowledge in the knowledge graph, but they cannot benefit from the large scale of labeled data. In other words, an exceptional specialist should process not just sound professional knowledge but also extensive experience.

For the TCM cerebral palsy diagnosis model to benefit from both the knowledge graph and the EMR, we propose a two-step model called KBRNN to achieve this purpose. In the first step, we extract evidence-based diagnostic knowledge from cerebral palsy KG by using intelligent optimization algorithms and represent this knowledge as tensors. Then, we inject the knowledge into RNN by converting the tensor to the parameter of the RNN. So far, we have obtained the knowledge-based RNN (KBRNN) that can be trained with the TCM data for fine-tuning.

Our key contributions are listed as follows:

1. We propose the knowledge-based RNN (KBRNN). Compared with the traditional methods, the KBRNN can be enhanced by the domain knowledge in KG. Also, the performance of KBRNN can be further enhanced by training on the labeled data.
2. Under the KBRNN proposed, we design an evolutionary algorithm for knowledge extraction and give an ingenious way to represent the knowledge as tensors and inject them into the RNN.
3. The experiment results show the accuracy of diagnosis of the untrained KBRNN which only with knowledge injections is 79.31%, and is up to 83.12% for the fully trained KBRNN.

## 2. Related Work

### 2.1. Knowledge Graph Inference and Its Applications

The knowledge graph contains the amount of prior knowledge [18], which can provide external information for various downstream tasks [19]. For medical tasks, Yang et al. [20] introduced the link prediction for the diagnosis of syndrome by dismantling medical records into multiple symptoms based on the KG. Zheng et al. [21] learned the relational embedding from nodes in KG to access medical knowledge and used them to improve the classifier's performance through the mechanism of medical knowledge attention. Zhang and Che. [22] constructed Parkinson's disease KG and KG completion methods that were leveraged to predict drug candidates. Yang et al. [23] pretrained the embeddings of entities by large-scale domain-specific corpus while learning the knowledge embeddings of entities via a joint TransC-TransE model. Lin et al. [24] combined the context provided by medical entity descriptions with the embeddings of medical entities and relations and user embeddings to learn patient similarities through a convolutional neural network. Lin et al. [25] utilized graph representation learning models to obtain the embedding vectors of the entities, then applied the embeddings to study patient similarities. These works used joint representation to bring entity and word vector space closer. However, for KGs with large numbers of entities, dealing with entities and their relationships leads to higher time complexity.

Furthermore, there is also some research about inference on the KG directly, without embedding the relations and entities. El-Shafai et al. [26] provided a method that simulates syndrome differentiation through Bayes and TF-IDF on a knowledge graph to achieve automated diagnosis in TCM. Yao et al. [27] presented an ontology-based model that utilized ontology attributes for training the neural network for medicine side-effect prediction. Xie et al. [28] applied the TF-IDF to the TCM KG and proposed a knowledge-based syndrome reasoning model.

### 2.2. Neural Network with Knowledge Enhance

Lin et al. [29] proposed a trigger matching network, which trains a trigger matching network with additional annotation and uses the output as the attention of the sequence labeler. Luo et al. [30] combined a neural network with regular expressions (RE) to improve supervised learning for natural language processing. Jiang et al. [31] proposed FA-RNN, a recurrent neural network that incorporates the benefits of both neural networks and regular expression rules. Finally, Jiang et al. [32] transformed regular expressions into neural networks to combine the two ways for slot filling.

## 3. Methods

### 3.1. Framework Overview

**Figure 1:**
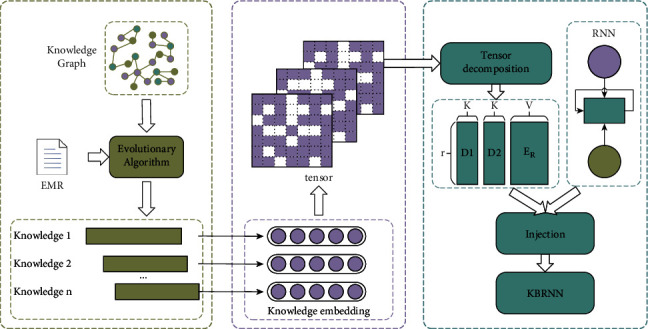
The model structure of KBRNN.

Figure 1 shows a two-step routine to construct a KBRNN, i.e., knowledge extracting and knowledge injecting. In knowledge extraction, an evolutionary algorithm is designed to extract high-scored knowledge from the KG. A part of EMRs is utilized to score knowledge. In knowledge injecting, knowledge is converted to a tensor in the knowledge embedding module. Then, the tensor decompose module decomposes the knowledge tensor as the parameters of RNN. This gives us the KBRNN which incorporates domain knowledge.

### 3.2. Notation

**Figure 2:**
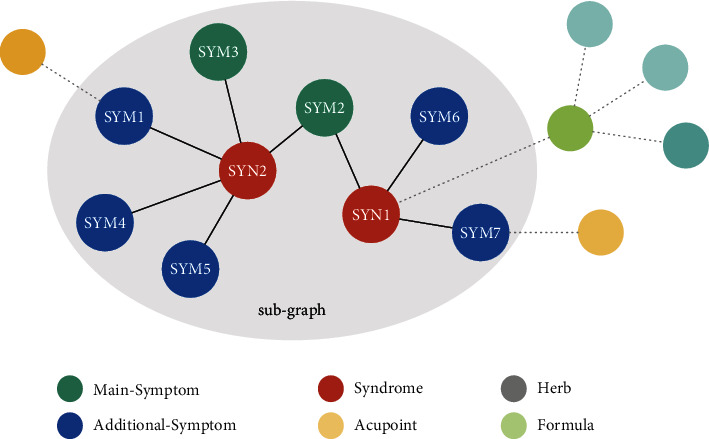
Structure of the knowledge graph.

To focus on diagnosing the syndrome by the patients' symptoms, shown in Figure 2, we reconstruct a sub-KG *K* based on the KG proposed by [33]. In this sub-KG *K* , we only retain the symptom and syndrome entities related to this research and exclude other entities such as acupoints, formula, and herb which are not related to diagnosis. For description, we give each symptom a unique and continuous ID starting from 0 and denote the symptom by “SYM”+ID. Similarly, we use “SYN”+ID to refer to a syndrome.

As a KG, *K* consists of entities *E* and relations *R*.

 
*E*: a set of entities. |*E*|=*N*. There are three types of entities (main symptom, additional symptom, and syndrome), *E*=*E*_main_sym_ ∪ *E*_add_sym_ ∪ *E*_syn_.
 
*R*: a set of relations. |*R*|=*M*.
 
*t*: Let *e*_*i*_, *e*_*k*_ ∈ *E*, *r*_*j*_ ∈ *R*, *t*=(*e*_*i*_, *r*_*j*_, *e*_*j*_) is the relationship between entities.

  In data processing and knowledge extraction, two common operations on *K* should be mentioned here.

 
*E*_Query(*SYNi*, *E*′): returns a set containing all the entities in *E*′ that are connected to *SYNi*.
 
*E*_match(sentence): sequential output the alias of entities which appear in sentence.

In this study, each EMR contains two parts: the descriptions of the main symptom and the additional symptom. Via data preprocessing, we splice the two parts of each EMR to get a sentence *s* and *c* convert each EMR to a symptom-level sentence by *E*_match(*s*). The EMR sentence corresponds to the EMR labeled as *SYNi* is defined as

(1) $$\begin{array}{c} s_{SYNi}=<{\text{sym}}_{1},{\text{sym}}_{2},\ldots {\text{sym}}_{i},{\text{sym}}_{i+1},\ldots {\text{sym}}_{n}>, \end{array}$$

where *s*_*SYNi*_[1, *i*]⊆*E*_main_sym_, *s*_*SYNi*_[*i* + 1, *n*]⊆*E*_add_sym_, *n* is the length of sentence *s*.

### 3.3. Extract Knowledge from KG

#### 3.3.1. Definitions and Task Complexity

This section details the thought to treat the knowledge extracting task as an optimization problem.

Above all, we define what the “knowledge” in the KG is. For the *SYN*2 shown in Figure 2, one of the knowledge about *SYN*2 denoted as Knowl_*SYN*2_ can acquire by (2) and the result as (3).

(2) $$\begin{array}{c} {\text{Knowl}}_{SYN2}=\left[E\_\text{Query}\left(SYN2,E_{\text{main\_sym}}\right),\left(E\_\text{Query}\left(SYN2,E_{\text{add\_sym}}\right)\right)\right], \end{array}$$

(3) $$\begin{array}{c} {\text{Knowl}}_{SYN2}=\left[\left[SYM3,SYM4,SYM5\right],\left[SYM2,SYM7\right]\right]. \end{array}$$

The (3) can be visually converted to a regular expression (RE) as (4), where “*|*” is the OR operator, “+” means one or more occurrences.

(4) $$\begin{array}{c} RE_{SYN2}={\left(SYM3|SYM4|SYM5\right)}^{+}{\left(SYM2|SYM7\right)}^{+}. \end{array}$$

Obviously, the sentence *s*_*SYN*2_ = <*SYM*5, *SYM*3, *SYM*7, *SYN*2> labeled as *SYN*2 can be recognized by *RE*_*SYN*2_. However, the risk raised with the *REs* is that it may lead to the wrong diagnosis. For example, *RE*_*SYN*2_ may also recognize the sentence *s*_*SYN*3_ = <*SYM*4, *SYM*2, *SYM*7> labeled as *SYN*3. For this issue, it looks like a feasible method that enumerates the subsets of *E*_main_sym_ and *E*_add_sym_, then, splicing them to generate Knowl_*SYN*_*i*__ as candidate solutions and filtering the useful Knowl_*SYNi*_ with the verification of EMR sentences for each syndrome. But the time complexity is as high as *O*(2^|*E*_main_sym_|^ × 2^|*E*_add_sym_|^) = *O*(2^|*E*_main_sym_|+|*E*_add_sym_|^) ≈ *O*(2^|*E*|^) = *O*(2^*N*^). Fortunately, too much knowledge injection complicates the diagnosis model, which will be discussed further in Section 3.4.2. Thus, for a specific syndrome named *SYNi* and a Knowl_*SYNi*_ scoring function *V*, it is enough to find the “top-*k *Knowl_*SYNi*_” corresponding to the *k* highest score Knowl_*SYNi*_ from  all the Knowl_*SYNi*_ of each syndrome.

**Figure 3:**
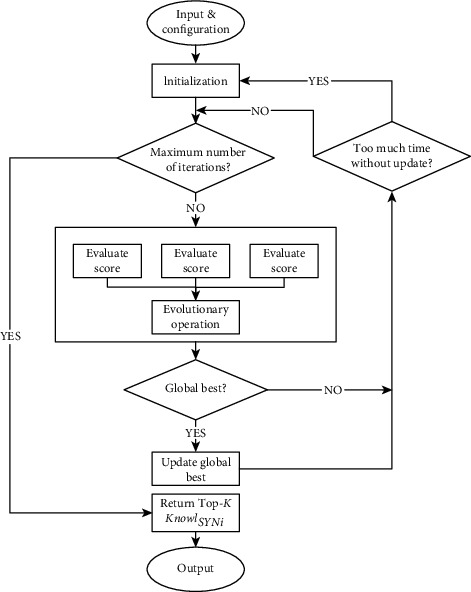
The steps of the evolutionary algorithm.

By the well-performance of the evolutionary algorithm in searching for relative optimal solutions from the large solution space, we design an evolutionary algorithm for knowledge extraction.  Figure 3 shows the main steps of the algorithm.

Our knowledge extraction method via evolutionary algorithms is based on the combination of two well-known expansions to the standard genetic strategy. On the one hand, we apply repeated reinitializations of the candidate solution when it reaches a state of stagnation. On the other hand, we utilize parallel computing in the process of evolution. While the former effectively overcomes the evolutionary algorithm's difficulty of falling into local optimal, the latter significantly improves the efficiency by allowing parallel calculation of the score of each solution. Moreover, assigning individuals to different computational cores can be viewed as a strategy for multiple population evolution, optimizing the algorithm's robustness.

There are two problem-specific modules in evolutionary algorithms, i.e., generator and evaluator. The following sections detail their specific implementation.

#### 3.3.2. Generator

The generator module creates the initial set of Knowl_*SYN*_*i*__ as candidate solutions for *SYNi*. A candidate solution corresponding to a Knowl_*SYNi*_ can be defined as a triple *τ*=<*φ*, *ψ*, *v*>, let |*E*_main_sym_ ∪ *E*_add_sym_|=*m*.

- *φ* ∈ {0,1}^*m*^: main symptom vector, *ϕ*[*j*]=1 if *SYMj* is selected, else *ϕ*[*j*]=0.
- *ψ* ∈ {0,1}^*m*^: additional symptom vector, *ψ*[*j*]=1 if *SYMj* is selected, else *ψ*[*j*]=0.
- *v* ∈ℝ: the score of such solution, calculated by using the evaluator module. Initialize to 0.

The generator generates a list of *τ*_*r*_=<*ϕ*_*r*_, *ψ*_*r*_, 0> denoted by Γ=[*τ*_0_, *τ*_1_, *τ*_2_,…, *τ*_*l*−1_] by initializing the *ϕ*_*i*_ and *ψ*_*i*_ randomly, where *l* is the length of Γ, *l* > *k*, 0 ≤ *r* < *l*.

#### 3.3.3. Evaluator

The evaluator calculates the score of each *τ* in Γ. We select *n* EMR sentences as the test case to compute the score of *τ*. This section will detail the scoring algorithm.

For a syndrome aliased *SYNi*, the evaluator divides the *n*  EMR sentences into two disjoint sets denoted by *EMR*_true_ and *EMR*_false_, where let |*EMR*_true_|=*a*, |*EMR*_false_|=*b*, *a*+*b*=*n*. A sentence is divided into *EMR*_true_ if and only if it is labeled as *SYNi*.

By these, variables *t*_*r*_, *f*_*r*_, *c*_*r*_ about solution *τ*_*r*_=<*φ*_*r*_, *ψ*_*r*_, *v*_*r*_> can be defined as follows:

- *t*_*r*_∈ℕ: the number of sentences in *EMR*_true_ which can be recognized by *τ*_*r*_
- *f*_*r*_∈ℕ: the number of sentences in *EMR*_false_ which can be recognized by *τ*_*r*_
- *c*_*r*_∈ℕ: the number of symptoms in *τ*_*r*_

As explained in Section 3.3.1, a high-score solution corresponding to an RE that recognizes the maximum number of *s*_*SYNi*_ while maintaining a minimal number of symptoms. In addition, misrecognition is not allowed. This provides us with the fundamental form of the scoring function equation.

(5) $$\begin{array}{c} V\left(\tau_{r}\right)=\frac{t_{r}}{c_{r}}\times 1_{N^{+}}\left(f_{r}\right), \end{array}$$

where 1_*N*^+^_() is the indicator function, 1_*N*^+^_(*f*_*r*_) = 1 if *f*_*r*_ ∈ *N*^+^, else 1_ℕ^+^_(*f*_*r*_)  =  0.

*c*_*r*_ can be calculated as equation.

(6) $$\begin{array}{c} c_{r}=\sum_{i=0}^{m-1}\phi \left[i\right]+\psi \left[i\right]. \end{array}$$

The following describes the calculation of *t*_*r*_ and *f*_*r*_. We maintain two matrices *TP*,  *FP* with the following rules.

- *TP* ∈ {0,1}^*a*×*m*^: *TP*[*i*][*j*]=1 if the *SYMj* in the i*th* sentence of *EMR*_true_, otherwise *TP*[*i*][*j*] = 0
- *TF* ∈ {0,1}^*b*×*m*^: *TF*[*i*][*j*]=1 if the *SYMj* in the i*th* sentence of *EMR*_false_, otherwise *TF*[*i*][*j*]=1

Then, we obtain *t*_*r*_ and *f*_*r*_ from equation (7) and equation (8), respectively.

(7) $$\begin{array}{c} t_{r}=\sum_{i=0}^{a-1}1_{N^{+}}\left\{\left[TP\left[i\right]-\left(\phi_{r}+\psi_{r}\right)\circ TP\left[i\right]\right]\times A^{T}\right\}, \end{array}$$

(8) $$\begin{array}{c} f_{r}=\sum_{i=0}^{b-1}1_{N^{+}}\left\{\left[FP\left[i\right]-\left(\phi_{r}+\psi_{r}\right)\circ FP\left[i\right]\right]\times A^{T}\right\}, \end{array}$$

where ∘ denotes element-wise product and *A* ∈ [1]^*m*^.

### 3.4. Convert the Knowledge to KBRNN

By the knowledge extraction algorithm details in Section 3.3, we get the top-*k *Knowl_*SYNi*_ for each syndrome. A Knowl_*SYNi*_ can be converted to an RE as (3)and (4) details in Section 3.3.1. We formally take the syndrome diagnosis task as a text classification problem, i.e., given an EMR sentence as the input of KBRNN, the output is the syndrome corresponding to the sentence.

For this task, as usual, we further process the EMR sentences as follows: we add the “BOS” and “EOS” at both ends of each EMR sentence as the mark of the start and end. We fill the sentence with “PAD”s to make all the sentences of the same length. Accordingly, to ensure that the RE corresponding to the Knowl_*SYNi*_ can recognize these new sentences, we add the $^*∗*^ at both ends of RE, while $ is the wildcard, and ^*∗*^ is the Kleene star operator. Take (3) as an example. The equation (4) corresponding to (3) can be rewritten as the following equation:

(9) $$\begin{array}{c} RE_{SYN2}=\text{\$}{\;}^{*}{\left(SYM3|SYM4|SYM5\right)}^{+}{\left(SYM2|SYM7\right)}^{+}\text{\$}* \end{array}$$

In the following section, we illustrate the implementation of the KBRNN, which is generated by injecting Knowl_*SYNi*_ into RNN.

#### 3.4.1. Embedding the Knowledge via Finite-State Automaton

Finite-State Automaton (FSA) is an abstract model of computation, which can change from one state to another in response to some inputs. The FSA can be used to recognize sentences. Given a sentence *s*=<′*BOS*′, sym_1_, sym_2_, sym3,…, sym_*n*_,′*EOS*′> , an FSA Λ, we feed the elements of *s* into Λ in order. Λ recognizes *s* if and only if the state transition sequence starts from the start state and ends with a final state.

There are two types of FSA: nondeterministic finite automaton (NFA) and deterministic finite automaton (DFA). The “deterministic” indicates that by giving the state an input, there is a unique transition to the next state. With Thompson's construction algorithm [34], an RE can be converted into an NFA. Then, the NFA can be converted to a unique DFA with a minimum number of states called m-DFA by the power set construction algorithm and the DFA minimization algorithm.

For *k* × |*E*_*syn*_|Knowl_*SYNi*_ obtained by the algorithm in Section 3.3, each Knowl_*SYNi*_ can be converted to an m-DFA. Then, we merge all the m-DFAs by adding a new start state *q*_*ϵ*_ and adding empty transitions from  *q*_*ϵ*_  to all start states of m-DFAs. This new FSA is denoted as *𝒜*, which can be defined formally as a 5-tuple: *𝒜*=<*Q*, Σ, *δ*, *q*_*ε*_, *F*′>.

 
*Q*: a nonempty, finite set of states. Let |*Q*|=*K*.
 
Σ: a nonempty, finite set of input vocabulary. Let |Σ|=*V*, *V* ∝ |*E*_*sym*_|.
 
*δ*: transfer function, *δ*(*q*, *σ*)=*p*(*p*, *q* ∈ *Q*, *σ* ∈ Σ).
 
*q*_*ϵ*_: the start state, *q*_*ϵ*_ ∈ *Q*.
 
*F*′: a nonempty, finite set of final states, *F*′⊆*Q*.

 Based on the above definition, we can represent *𝒜* equivalently by matrixes *T*,  *S*,  *F*.

 
*T* ∈ {0,1}^*V*×*K*×*K*^: the transfer matrix, *T*[*σ*, *i*, *j*]=1 if the state *q*_*i*_ can transit to *q*_*j*_ when input a vocabulary *σ*, otherwise 0. (*q*_*i*_, *q*_*j*_ ∈ *Q*, *σ* ∈ Σ).
 
*S* ∈ {0,1}^*K*^:  *S*[*i*]=1 if *q*_*ϵ*_ can transit to *q*_*i*_ directly, otherwise 0.
 
*F* ∈ {0,1}^*K*^: *F*[*i*]=1 if *q*_*i*_ ∈ *F*′, otherwise 0.

Now, we obtain the knowledge embedding 〈*T*,  *S*,  *F*〉.

#### 3.4.2. Inject the Knowledge Embedding into RNN

For a sentence *s*= <*s*_1_, *s*_2_, *s*_3_ …, *s*_*x*_>, the Out(*s*) denotes the number of *s* recognized by m-DFAs, which can be expressed as the following equation:

(10) $$\begin{array}{c} \text{Out}\left(s\right)=S^{T}\cdot \left(\overset{x}{\underset{i=1}{\prod }}T\left[s_{i}\right]\right)\cdot F. \end{array}$$

Here, we extend the approach in [31], which used canonical polyadic decomposition (CPD) to decompose *T* into *E*_*R*_ ∈ *R*^*V*×*r*^, *D*_1_ ∈ *R*^*K*×*r*^, *D*_2_ ∈ *R*^*K*×*r*^, where *r* is a hyperparameter. As the study in [31], the decomposition is approximate when the *r* converges to the rank of *T*, and if *r* is too large, it may lead to a higher space complexity. In this work, the rank of *T* is positive to the number of symptoms in Knowl_*SYNi*_. That is why, we must maintain a minimum number of symptoms in Knowl_*SYNi*_.

*E*_*R*_ has a dimension equal to the size of the input set *Σ*, which can be considered as the word embedding of each input word. In this work, we integrate the BERT [35] embedding into *E*_*R*_. Let *w*_*t*_ be the word embedding of *s*_*t*_, *u*_*t*_ be the 768-dim word embedding generated by using bert-base-chinese, and *v*_*t*_ be the embedding of *s*_*t*_ in *E*_*r*_. The BERT embedding can be integrated by equation (11). Here, *β* ∈ [0,1] is a hyperparameter, and *G* ∈ *R*^*D*×*r*^ is a trainable matrix.

(11) $$\begin{array}{c} \;w_{t}=\beta v_{t}+\left(1-\beta \right)u_{t}G. \end{array}$$

With the CPD result, the equation (10) can be rewritten to the recurrent form similar to the formal definition of RNN as the following equation:

(12) $$\begin{array}{c} a=\left(h_{t-1}\cdot D_{1}\right)\circ w_{t},h_{0}=S,\\ h_{t}=a\cdot D_{2}^{T},\\ \text{Out}\left(s\right)=h_{x}\cdot F. \end{array}$$

So far, we have obtained the RNN injected with knowledge.

## 4. Experiments and Results

### 4.1. Datasets

We collect the dataset from a project by the National Key Research and Development Program of the Chinese Academy of Traditional Chinese Medicine, “Chinese Medicine Data Center and Health Cloud Platform Building.” The EMR data are mainly from the Hospital Information System (HIS), which includes admission records, course records, discharge summaries, and medical records of cerebral palsy patients within a specific time frame. These data come from clinically valid cases and have been desensitized to protect patients' private information.

The original EMR data has several flaws, including a nonstandard format and diverse expression. A team of professionals is invited to tag the EMR data manually so that it may be organized into structured data for further research. Data tagging assumes the form of two-person cooperation to prevent errors caused by the limited expertise of a single individual. There remain nonstandard data in the structured data after the data tagging process. For instance, a particular symptom may have several distinct expressions. In data standardization, numerous professional words are first standardized and sorted out collaboratively by a group of individuals. Then, a medical specialist induces the standard terms included in the medical records. In the end, the standardization of 988 symptoms and 15 syndromes was achieved. According to traditional Chinese medicine, these symptoms may be further subdivided into main symptoms and additional symptoms. The main symptoms might generally represent the patients' overall condition, but the additional symptoms relate to complications, which is a significant diagnostic criterion for syndrome kinds.

Thus, we obtained 5514 labeled diagnostic records from 1755 patients. Each record has three fields, main symptoms, additional symptoms, and syndrome as the label.

### 4.2. Experimental Steps

We divide the EMR dataset randomly into the following four parts:

- Pre-set (20%): the pretrained dataset that engages in the scoring of knowledge in the knowledge extraction algorithm
- Train-set (50%): train dataset, the dataset used for training models
- Dev-set (20%): validation dataset, a set of examples used to tune hyperparameters
- Test-set (10%): test dataset, a dataset for testing the performance of the trained model

During the knowledge extraction phase, we execute the evolutionary algorithm and utilize pre-set data for knowledge scoring and obtain top-*k *Knowl_*SYNi*_ (*k* = 6 in practice) for each syndrome. We removed some Knowl_*SYNi*_ that scored poorly, which is caused by the insufficiency of the corresponding syndromes' sample sizes.

During knowledge embedding and injecting, we obtain an untrained KBRNN that has not been trained on the train-set. We adopt some conventional machine learning models which are frequently used in text classification as baselines and compare them with KBRNN. For each baseline, we feed the hidden representation produced by these models into a multilayer perceptron (MLP) and use the cross-entropy loss as the objective function.

### 4.3. Experimental Results

We compare KBRNN with RNN [36], LSTM [37], GRU [38], 4-layer CNN [39], 4-layer DAN [40] as well as their bidirectional variants. We use the cross-entropy loss as the objective function and input the hidden representation generated by these models into a 3-layer MLP to obtain the label logits. For each dataset, we tune the learning rates from [0.01, 0.005, 0.001, 0.0005, 0.0001] and the number of hidden states in [50,  100,  150,  200]. Two potential benefits are explored as follows:

1. The contribution of knowledge extraction to KBRNN: we use the pre-set as the training set of baselines and compare the performance of untrained KBRNN and baselines on the test set
2. The ability of KBRNN to benefit from labeled data: we utilize both the pre-set and the train-set (50%, 100%) as the training set and fine-tune the untrained KBRNN with the train-set

Table 1 displays the classification accuracy of the KBRNN and baseline models on the test-set after training with varying amounts of training data. The KBRNN can achieve 79.31% diagnostic accuracy with only injecting the knowledge extracted from the KG based on pre-set and rises to 83.12% with sufficient training based on the 100% train-set.

**Figure 4:**
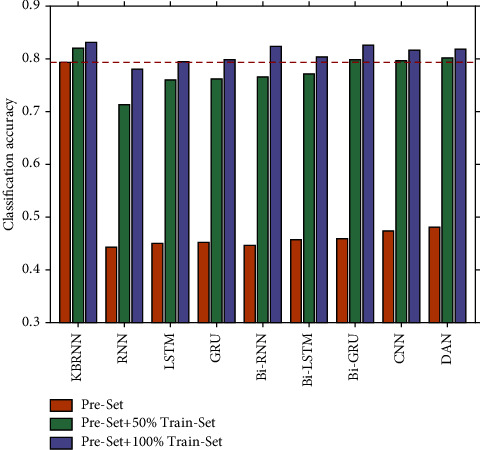
The untrained KBRNN outperforms some of the full-trained baselines.

**Figure 5:**
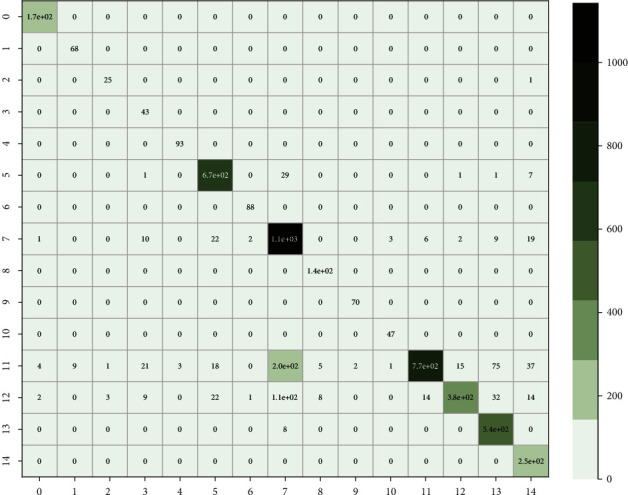
Confusion matrix of the classification result by using the fully trained KBRNN for all the samples.

The result shows that the untrained KBRNN outperforms all the other baselines which are only trained on the pre-set. It is also better than some of the baselines trained with 50% of the train-set (Figure 4.). We believe that KBRNN obtains considerable a priori knowledge from the knowledge graph through injection. The classification result on the full samples by using the fully trained KBRNN is shown as the confusion matrix in Figure 5, which provides a good insight into how often samples of each fifteen syndromes are correctly classified or misclassified by the proposed model. We can find that the number of samples varies greatly in each syndrome type, and the true positive rate could be maintained at a high level even for the syndrome with a large number of samples. As with other models, KBRNN can benefit from expanding the training set while keeping accuracy benefits.

## 5. Discussion and Conclusions

TCM, as a complementary field of medicine outside the modern medicine system, has played a significant role in cerebral palsy syndrome diagnosis. In this work, we propose a knowledge-based RNN (KBRNN) for cerebral palsy syndrome diagnosis. Our major contribution is building an evolutionary algorithm to extract the diagnosis knowledge from the KG. In particular, we also present the method of injecting the TCM knowledge into the RNN. Compared with the simple KG inference or the rule-based methods, as a neural network model, the KBRNN can be further trained by EMR data, which makes the KBRNN more generalized. On the other hand, compared with the traditional neural network model, KBRNN can benefit from TCM knowledge. Specifically, with the help of TCM knowledge, KBRNN outperforms previous neural approaches in the scene where only a few EMRs are available, and it remains competitive in rich-resource settings.

In conclusion, KBRNN can benefit from two aspects, i.e., knowledge extracted from the cerebral palsy knowledge graph and labeled EMR. We show that KBRNN achieves higher accuracy in syndrome diagnostic tasks only with knowledge injection. Moreover, the performance of KBRNN can be further improved after training with a large amount of labeled EMR, which outperforms the current model.

## Figures and Tables

**Table 1:** The classification accuracy of the KBRNN and baselines.

	Preset	Preset + 50% train-set	Preset + 100% train-set
KBRNN	79.31	82.03	83.12
RNN	44.28	71.32	78.03
LSTM	45.01	76.04	79.49
GRU	45.19	76.22	79.85
Bi-RNN	44.64	76.59	82.39
Bi-LSTM	45.74	77.13	80.40
Bi-GRU	45.91	79.85	82.58
CNN	47.37	79.67	81.67
DAN	48.09	80.21	81.85

## Data Availability

All data included in this study are available upon request by contact with the corresponding author.

## References

[1] Graham H. K., Rosenbaum P., Paneth N. (2016). Erratum: cerebral palsy. *Nature Reviews Disease Primers*.

[2] Zhou X., Menche J., Barabási A.-L., Sharma A. (2014). Human symptoms–disease network. *Nature Communications*.

[3] Wang Y., Shi X., Li L., Efferth T., Shang D. (2021). The impact of artificial intelligence on traditional Chinese medicine. *The American Journal of Chinese Medicine*.

[4] Zhou X., Chen S., Liu B. (2010). Development of traditional Chinese medicine clinical data warehouse for medical knowledge discovery and decision support. *Artificial Intelligence in Medicine*.

[5] Wang H. (2008). A computerized diagnostic model based on naive bayesian classifier in traditional Chinese medicine. *International Conference on BioMedical Engineering and Informatics*.

[6] Zhang D., Pang B., Li N., Wang K., Zhang H. (2005). Computerized diagnosis from tongue appearance using quantitative feature classification. *The American Journal of Chinese Medicine*.

[7] Hu X., Zhu H., Xu J., Xu D., Dong J. Wrist pulse signals analysis based on deep convolutional neural networks.

[8] Fu S., Zheng H., Yang Z. Computerized tongue coating nature diagnosis using convolutional neural network.

[9] Hou J., Su H.-Y., Yan B. Classification of tongue color based on cnn.

[10] Yang K., Zheng Y., Lu K. (2022). PDGNet: predicting disease genes using a deep neural network with multi-view features. *IEEE/ACM Transactions on Computational Biology and Bioinformatics*.

[11] Dai Y., Wang G., Dai J., Geman O. (2020). A multimodal deep architecture for traditional Chinese medicine diagnosis. *Concurrency and Computation: Practice and Experience*.

[12] Liang Z., Liu J., Ou A., Zhang H., Li Z., Huang J. X. (2019). Deep generative learning for automated EHR diagnosis of traditional Chinese medicine. *Computer Methods and Programs in Biomedicine*.

[13] Sung S.-F., Lin C.-Y., Hu Y.-H. (2020). EMR-Based phenotyping of ischemic stroke using supervised machine learning and text mining techniques. *IEEE Journal of Biomedical and Health Informatics*.

[14] Chen X., Jia S., Xiang Y. (2020). A review: knowledge reasoning over knowledge graph. *Expert Systems with Applications*.

[15] Ji S., Pan S., Cambria E., Marttinen P., Yu P. S. (2022). A survey on knowledge graphs: representation, acquisition, and applications. *IEEE Transactions on Neural Networks and Learning Systems*.

[16] Gong F., Wang M., Wang H., Wang S., Liu M. (2021). SMR: medical knowledge graph embedding for safe medicine recommendation. *Big Data Research*.

[17] Abdelaziz I., Fokoue A., Hassanzadeh O., Zhang P., Sadoghi M. (2017). Large-Scale structural and textual similarity-based mining of knowledge graph to predict drug-drug interactions. *Journal of Web Semantics*.

[18] Liu W., Zhou P., Zhao Z. (2020). K-BERT: enabling language representation with knowledge graph. *Proceedings of the AAAI Conference on Artificial Intelligence*.

[19] Wang H., Ren H., Leskovec J. (2021). Relational message passing for knowledge graph completion. https://arxiv.org/abs/2002.06757.

[20] Yang R., Ye Q., Cheng C., Zhang S., Lan Y., Zou J. (2022). Decision-making system for the diagnosis of syndrome based on traditional Chinese medicine knowledge graph. *Evidence-based Complementary and Alternative Medicine*.

[21] Zheng W., Yan L., Gou C. (2021). Pay attention to doctor–patient dialogues: multi-modal knowledge graph attention image-text embedding for COVID-19 diagnosis. *Information Fusion*.

[22] Zhang X., Che C. (2021). Drug repurposing for Parkinson’s disease by integrating knowledge graph completion model and knowledge fusion of medical literature. *Future Internet*.

[23] Yang Y., Yin X., Yang H. (2021). *KGSynNet: A Novel Entity Synonyms Discovery Framework with Knowledge Graph*.

[24] Lin Z., Yang D., Yin X. (2020). Patient similarity via joint embeddings of medical knowledge graph and medical entity descriptions. *IEEE Access*.

[25] Lin Z., Yang D., Jiang H., Yin H. (2021). Learning patient similarity via heterogeneous medical knowledge graph embedding. *IAENG International Journal of Computer Science*.

[26] El-Shafai W., A Mahmoud A., M El-Rabaie E. S. (2022). Traditional Chinese medicine automated diagnosis based on knowledge graph reasoning. *Computers, Materials and Continua*.

[27] Yao Y., Wang Z., Li L. (2019). An ontology-based artificial intelligence model for medicine side-effect prediction: taking traditional Chinese medicine as an example. *Computational and Mathematical Methods in Medicine*.

[28] Xie Y., Hu L., Chen X., Feng J., Zhang D. (2020). Auxiliary diagnosis based on the knowledge graph of TCM syndrome. *Computers, Materials and Continua*.

[29] Lin B. Y., Lee D.-H., Shen M. (2020). TriggerNER: learning with entity triggers as explanations for named entity recognition. https://aclanthology.org/2020.acl-main.752.

[30] Luo B., Feng Y., Wang Z. (2018). Marrying up regular expressions with neural networks: a case study for spoken language understanding. https://arxiv.org/abs/1805.05588.

[31] Jiang C., Zhao Y., Chu S., Shen L., Tu K. (2020). Cold-start and interpretability: turning regular expressions into trainable recurrent neural networks. https://aclanthology.org/2020.emnlp-main.258.

[32] Jiang C., Jin Z., Tu K. (2021). Neuralizing regular expressions for slot filling. https://aclanthology.org/2021.emnlp-main.747.

[33] Mou Z. (2021). Study on the construction of tcm diagnosis and treatment knowledge map and the dominance of tacit knowledge in children with cerebral palsy. *Chinese Academy Of Traditional Chinese Medicine*.

[34] Thompson K. (1968). Programming Techniques: regular expression search algorithm. *Communications of the ACM*.

[35] Devlin J., Chang M.-W., Lee K., Toutanova K. (2019). Bert: pre-training of deep bidirectional transformers for language understanding. https://arxiv.org/abs/1810.04805.

[36] Elman J. L. (1990). Finding structure in time. *Cognitive Science*.

[37] Hochreiter S., Schmidhuber J. (1997). Long short-term memory. *Neural Computation*.

[38] Chung J., Gulcehre C., Cho K., Bengio Y. (2014). Empirical evaluation of gated recurrent neural networks on sequence modeling. https://arxiv.org/abs/1412.3555.

[39] Kim Y. (2014). Convolutional neural networks for sentence classification. https://arxiv.org/abs/1408.5882.

[40] Iyyer M., Manjunatha V., Boyd-Graber J., Daumé H. (2015). Deep unordered composition rivals syntactic methods for text classification. https://aclanthology.org/P15-1162.

